# Transactions of the Chicago Gynæcological Society

**Published:** 1886-06

**Authors:** 

**Affiliations:** 2330 Indiana Ave.


### SOCIETY REPORTS.

TRANSACTIONS OF THE CHICAGO GYNÆCOLOGICAL SOCIETY.

*LXIV Meeting, Friday, March 19th, 1886.*

*I.—Presentation of Instruments and Pathological Specimens.*

*II.—Adjourned Discussion of Pelvic Cellulitis.*

*III.*—MILLER. *Report of a Case of Epithelioma of the Uterus; Treated by Mercuric Nitrate; Result Six Years After Treatment.*

### I.—THE PRESENTATION OF INSTRUMENTS AND PATHOLOGICAL SPECIMENS.

PROFESSOR W. W. JAGGARD wished to call attention to a new instrument and a pathological specimen, in order to remind the Fellows of the Society of the first regular order of business, and on account of the intrinsic interest the instrument and the specimen possessed.

*1.—Professor Alex. J. Stone's Pelvic-outlet Forceps.*

Professor Alex. J. Stone, of St. Paul, has designed a very valuable pelvic-outlet forceps.

The characters of the instrument are:

Length, 24 cm. (Spoons, 14 cm., handles, 10 cm.)

Fenestra, length, 12 cm., width, 3.5 cm.

Cephalic curve, 8 cm.

Weight, 210 grammes.

English lock.

The instrument is bent backwards on its long axis, so that the angle, at the junction of the spoons and handles, is about 160°. The object of the perineal bend is to maintain flexion of the head during its passage through the vulvar orifice.

PROFESSOR JAGGARD had recently employed Professor Stone's forceps in a case in which the head was arrested at the vulvar orifice, the result of uterine inertia caused by a large intra-mural fibroid. It was possible to apply the forceps in the left lateral position without the patient's knowledge. He thought the instrument possessed obvious advantages over Dr. Sawyer's excellent pelvic-outlet forceps.

2.—*Puerperal Uterus and Adnexa.*

The pathological specimen was the uterus and adnexa, removed from the body of a patient who died at the Cook County Hospital on the third day of the *puerperium.* Dr. H. H. Frothingham, one of the resident obstetricians, had kindly placed the material at the speaker's disposal. The patient, thirty years old, multipara, was admitted to the Hospital on the thirteenth of November, 1885, and gave a history of forceps delivery of a dead child, with perineal laceration, on the eleventh of November. Temperature, 103.4° F.; pulse, weak and rapid; respiration, shallow and frequent; tongue, dry and brown; pulmonary œdema; rigors; profound prostration.

Abdominal tenderness, tympanites, dullness in both flanks; two tumors, the size of a hen's egg, on either side of the vulvar orifice; *labia majora* apparently gangrenous; recent perineal laceration; foul odor from vagina; complete cessation of lochial secretions.

The patient died soon after admission to the Hospital.

*Autopsy.*—Both pleural cavities about half filled with seropurulent fluid and flakes of lymph; lungs œdematous; pericardium contained three ounces of fluid similar to that within the pleuræ; *endocardium apparently normal*; myocardium soft and friable; no metastatic abcesses could be found. Peritoneal cavity contained about one gallon of sero-purulent fluid, with flakes of lymph; intestines contracted, but no adhesions; liver enlarged, congested, giving evidence of extensive fatty degeneration; *spleen*, of *normal size;* kidneys, cortex giving evidence of fatty degeneration, pelves injected and intensely hyperæmic; peritoneum injected.

The uterus was of a size, corresponding to the third day of the *puerperium*. The ring of Bandl was plainly demonstrable. The endometrium exhibited the pseudo-membranous necrosis, clearly described by Birch-Hirschfeld and and other pathologists by the term *endometritis diphtheritica.*

The mucous membrane of the vagina showed similar diphtheritic changes. Pus oozed through both Fallopian tubes. The left ovary and tube were intensely injected. Puerperal ulcers were visible on either side of the vulvar orifice. The tumors on either side of the vulvar orifice proved to be caused by hæmorrhage into the perivaginal connective tissue, —pudendal hæmatomata. No examination for micro-organisms was made.

DR. JAGGARD thought the case was a typical example, both as regards the clinical course and anatomical findings, of that form of puerperal fever, described by Buhl in 1861, as *Puerperal Fever without Pyæmia*, (endocolpitis, endometritis, salpingitis, peritonitis—subsequently pleuritis and pericarditis.) The entire absence of splenic tumor was worthy of particular notice. Carl Braun has justly attached great significance to this sign,—even going so far as to call it pathognomonic. The apparently normal state of the endocardium was remarkable.

II.—ADJOURNED DISCUSSION OF PELVIC CELLULITIS.

PROFESSOR A. REEVES JACKSON stated that he understood from the notice received that he was expected to introduce the general subject of pelvic cellulitis. If anything should cause a sense of humiliation to come to us as medical men it was the impression which was forced upon us of how little accurate knowledge we had upon even the most ordinary topics when we attempted to study any medical subject in detail. In order to introduce the subject of pelvic cellulitis he should say at least something about its pathology, diagnosis and treatment. In regard to the first of these subjects, Professor Thorburn says that he inquired of several experienced anatomists whether they knew of the existence of cellular tissue in the broad ligaments, and they replied that they did not know of it except from current gynæcological literature; of course that was because they had not investigated the subject practically; we do know there is a substance called cellular tissue in the pelvis, that it exists in and between folds of the peritoneum forming the broad ligaments between the uterus and the bladder, and also posteriorly to the uterus. When we speak of cellulitis we mean an inflammation affecting this tissue and all that is connected with it; that is, its lymphatics, glands, blood-vessels and nerves. Some think the starting point is nearly if not always in the lymphatic glands, so that here in the outset we are met with this evidence of lack of certain knowledge. He thought it did not matter much to us as practitioners whether that layer called cellular tissue is affected in one portion only or whether other structures are also involved. The diagnosis presents difficulties, as we all know. In the first place, we do not always know when the disease is present, and in every stage we feel doubt as to whether this disease or some other is present; sometimes even when it goes on to its later stages we have doubt. The first evidence of this disease consists in an effusion of serum which produces a hardness in the part and which becomes more and more marked as the disease progresses; this may be of longer or shorter duration; it may disappear by absorption; or it may not disappear at all, but go on until the effusion becomes pus, forming pelvic abscess. Pelvic cellulitis and pelvic peritonitis frequently exist together, and as it does not affect our treatment of the case in the early stages this fact does not make much difference to us as practitioners. Coming to the question of treatment, he had always felt a great deal of doubt as to the efficacy of the usual means employed—opium, digitalis, quinine, etc.; that is, as to whether they have any controlling influence upon the progress of the disease. From the very nature of the inflammatory process he scarcely thought these things do more than simply palliate the symptoms. Prolonged hot water douches are doubtless remedial, and may abort the disease. Latterly, interest has been more centered in the treatment of the condition when it has advanced to the stage of abscess. Heretofore the treatment has been notoriously unsatisfactory, so that cases have gone on for weeks, months and years, the woman being constantly subject to discharges of pus, and the remedial means have done little more than assist nature in the escape of the fluid. But during the last few years, since attempts have been made to treat the disease radically by surgical means, there has been offered an additional and efficacious method of dealing at least with some of these cases. He alluded to laparotomy. The success that has been attained in this way should lead us to look upon it with favor. He thought, however, that where radical surgical means are resorted to there is danger of too frequently performing operations dangerous in themselves, to relieve a disease which perhaps would not end life, although rendering the patient an invalid during her lifetime. He believed the discussion would be profitable if it should largely take the course of considering this latest and most formidable measure of treatment. He had read the account of Prof. Fenger, giving an account in detail of three operations made by himself; two of the operations were followed by death, which was attributed to some other disease, co-existent perhaps with the abscess itself; the other was successful. You are familiar with Lawson Tait's treatment for these encysted collections of pus. His success is simply marvelous. In 1883 the *British Medical Journal* published an account of twenty-four cases in which he had been successful, and in a letter which Dr. Jackson had received from him recently he said that up to that time he had operated successfully on thirty-two cases.

This was all the introduction Professor Jackson had to offer, and it opened the whole subject. He was reminded that he had not stated whether he approved of abdominal section for this cause. He said he did, unhesitatingly, and thought there was as good reason for operating in inflammation of the pelvis as in any other disease which produces lifelong invalidism, provided it were not curable by other means

Laparotomy offers a method which is perhaps applicable to a comparatively small number of cases, and yet here it is the only remedy, that is, in cases of long standing in which the abscess cavity cannot be otherwise reached for the purpose of drainage, and where the women is of such an age that makes it reasonable to suppose she will suffer for many years. He considered it a dangerous operation, and there had been errors in diagnosis. Mr. Tait is justified in his bold method of diagnosticating abdominal disease by laparotomy. He follows it up with removal of the disease. Where the diagnosis is fairly established he believed that laparotomy is a proper procedure as a last resort.

PROFESSOR W. H. BYFORD thought there was not much more to be said than had been said by Professor Jackson, who had given an admirable *résumé* of the subject in all its bearings in such a way as to set it before the Society with clearness, and he approved of all Professor Jackson had said, without exception. Professor Byford had already said to the Society what he had to say upon the subject of pelvic abscess, and what he thought of the conditions of the operation of laparotomy for the abscess. He could not get rid of the idea that the operations performed by Lawson Tait were cases in which the abscesses were largely in the abdominal cavity, and were not pelvic abscesses, properly speaking—certainly were not confined to the pelvis; and he believed from what he had seen in regard to his cases that almost all were encysted peritoneal, instead of encysted pelvic abscesses. In speaking upon the subject before, he had taken the stand that the operation of cutting into the peritoneal cavity for the purpose of going down into the pelvis when the abscess did not reach above the pelvic rim, was not justifiable in many instances, if at all. Where there has been a bar thrown out by the effusion of lymph in the peritoneal cavity so as to isolate the purulent collection and it extends up into the abdominal cavity, then he had no doubt of the propriety of the supra-pubic operation. He had recently seen an abscess, which formed mainly above the pelvic brim, open into the rectum at the top of the pelvis, taking its course above and across the *fundus uteri* and opening in the side of the rectum. The collection of pus was entirely above the pelvis, and it was necessary to cut through the muscles and fascia to get into the pus cavity and then establish a process of drainage through the rectum. It would have been impossible in that case to have effected the opening through the vagina.

Professor J. H. Etheridge said he had had no experience in opening the abdomen for treatment of abscess in the pelvis. He had seen one abscess opened through the abdominal wall, which had a spontaneous opening into the rectum. It was done at the Presbyterian Hospital about a year ago. The top of the abscess could be felt through the wall, and an attempt was made to find the opening, wash it out and inject it; but this being regarded as impracticable, abdominal section was decided upon, and a very curious combination of pathological conditions was discovered. The abdomen was opened, and a protruding something was discoverable; there was a discharge of perhaps an ounce and a half of serum, then it was cleaned. It was really a cystic growth, and upon introducing the finger another cyst, apparently, was discovered, that was opened and thoroughly evacuated, then another protruding by it was discovered, which was found to be the abscess cavity itself. That was opened and cleaned out and a drainage tube introduced and fastened to the wound, and the patient dressed, but she died within twenty-four hours. Post-mortem examination was made, and the track of the opening readily marked out. He had seen pelvic abscesses that opened in all directions through the abdominal cavity, into the bladder, vagina, and rectum, but never saw one that opened into the uterus. He saw at the Woman's Hospital an abscess that opened through the vagina and abdominal wall. The patient died. Dr. Etheridge said he had under treatment a pelvic abscess that opens into the rectum, in which general cellulitis is present. About once in six weeks the woman would have a fever, and after undergoing a great deal of suffering, nights of pain in which she would have to take large quantities of opium, there would be a discharge of pus into the rectum. She was put on tonics, and her general health improved greatly. Now she goes a great many weeks at a time without any return of the symptoms. A slight discharge of pus is present with every discharge from the bowels. It seemed to him as if the true estimate to make of the justifiability of laparotomy for abscesses is the ability to reach the top of the abscess through the abdominal wall, and where it cannot be reached it seemed to him that purulent discharge of the abscess into the abdominal cavity would surely take place.

DR. EDWARD WARREN SAWYER thought it a matter of regret if the discussion were to conclude without the special indications being given for a line of treatment in given cases. For his own satisfaction, he would like to have an answer to the following questions: First, in case of pelvic abscess, in which there is already an opening established, either by the bowel or by the bladder, is laparotomy ever justifiable? Second, in a case of supposed pelvic suppuration, without any external manifestation, as an opening through the bowels or bladder, is laparotomy ever justifiable?

DR. A. H. FOSTER said he had not been present long enough to learn the scope of the discussion, and had but little to add. He did not recollect any case of pelvic abscess in his experience, although it was that of constant care of cases of pelvic cellulitis, more or less extensive. He always expected to have a larger crop of such cases, of more or less gravity, at this time of the year than at any other, especially those cases that are non-puerperal, but are menstrual. The cause seemed to be carelessness in regard to clothing, especially of the feet, and the exposure which comes from the varying weather in this climate, hot and cold, wet and dry. In regard to treatment of these cases, his treatment did not differ from others. He always relied on prompt doses of mercury and opium until the acute symptoms subsided; fomentations externally and hot douches internally, and then followed either with the muriate of ammonium or the iodide of potassium, reducing the opiate as fast as possible and continuing quinine or its substitute.

DR. PHILIP ADOLPHUS said that in the absorption of recent inflammatory deposits in the pelvis, he depended on the action of the skin, kidneys and bowels, stimulated by proper medication. In regard to hot douches, it depended whether the surroundings of the patient are such that they can be given properly; if otherwise, to attempt it will result injuriously. If symptoms point to suppuration, and fluctuation can be felt *per vaginam*, the use of the hypodermic syringe will disclose serum or pus. If serum, he trusted to absorption; pus, however, necessitated a speedy incision, with subsequent drainage, etc. If formation of pus is suspected at any time, it is best to look for it, and if it cannot be found, it is best to wait for its pointing. However, if the patient is in great danger, laparotomy is indicated.

DR. F. E. WAXHAM was sorry that he had not been able to hear the whole discussion. He was reminded, quite forcibly, of a laparotomy upon one of his own patients; however, it was not a case of pelvic cellulitis—it was acute peritonitis, with effusion within the abdominal cavity. This patient presented all the characteristic symptoms of peritonitis, intense abdominal pain, exceeding tenderness and great abdominal distension; the patient passed from bad to worse, and finally fluctuation was detected in the lower portion of the abdominal cavity. An eminent surgeon was called in consultation, who recommended laparotomy as the only hope of saving the patient's life. It was supposed there was pus within the abdomen, and, although very feeble and greatly exhausted, it was considered proper to give the patient the benefit of the operation. Frequent hypodermic injections of brandy were given, in order to support the flagging pulse during the operation. The abdomen was opened, but no pus was discovered in the abdominal cavity; a thin, serous fluid was discovered. The patient barely survived the operation, dying about six hours later from prostration and shock. In contrast to this patient, he remembered another—a case of peritonitis in a child—that had all the characteristic symptoms of acute, violent peritonitis. This patient was seen by Dr. W. H. Byford, who gave valuable assistance in the treatment. The child, who was but seven years old, was obliged to take twenty drops of tincture of opium every hour and a half for two days at a time, in order to control the intense pain, and it seemed as if it was impossible for it to live from one hour to another; fluctuation was found more marked in this case than in the other, but, notwithstanding all these unfavorable symptoms, this little patient made a gradual, slow, tedious, but yet permanent recovery. He believed we ought not to subject a person to so dangerous an operation as laparotomy without proof of the presence of pus. It seemed to him that a serous fluid might become absorbed and the patient recover, where an operation would very likely cause death.

PROFESSOR CHARLES WARRINGTON EARLE said he had nothing new to add in regard to the ordinary general treatment of pelvic cellulitis or abscess, but he was practically interested in exactly the questions that had been asked by Dr. Sawyer. He spoke of the terrible cases which he supposed all physicians meet occasionally. He had probably seen half a dozen during his fifteen years of practice. These cases have a history of pelvic pain and inflammation, and finally a discharge of pus, which continues for weeks and months, and sometimes years. At last some of us are called in and find that the woman is suffering from chills, a high temperature, feeble pulse, and is greatly exhausted. He wanted the older members to tell what should be done with such cases. Is there any hope for a woman in this condition? A few months ago he was called in consultation with another physician to see a lady who had a constantly discharging abscess, until she was nearly dead from exhaustion. The question in his mind was,—and it was one he would like to have discussed,—will it do any good to open the abdomen of such a woman and clean out those pus pockets? Have we any experience which will give us a moderately safe rule by which to proceed in such a case, where death is absolutely certain if something out of the usual line is not done? He hoped some light would be thrown on this question.

DR. HENRY T. BYFORD thought that the treatment of the first stage had been spoken of too slightly. He had seen much irreparable harm done by the failure of physicians to promptly recognize and properly treat the acute attacks. There is no doubt of the positive influence of opium on the peritoneal part of the inflammation, of sedatives on the arterial excitement, of absolute quiet of body (opium acts largely by quieting all motion in the bowels), and of the local application of heat. But he protested against the early indiscriminate use of the vaginal douche. He had known it to do decided harm.

He attributed the lack of hopefulness, in speaking of the management of suppurating cases, to the lingering influence of past experience. In the light of our present knowledge, the lines can be drawn much more definitely. A pelvic abscess should of course be opened wherever it may point, whether in the vagina, rectum, or under the skin. When the primary outlet is made though the skin, the treatment, being entirely antiseptic in character, leaves nothing to be desired; when made through the vagina or rectum, the outlet must be, as nearly as practicable, at the most dependent portion, for strict antisepsis being impossible, drainage should be perfect. Spontaneous primary openings through the cutaneous surface that do not admit of a thoroughly antiseptic management must be modified so as to admit of it; and openings through the vaginal or rectal walls, that do not secure adequate drainage, must be made to do so. The most troublesome of these abscesses are those that have formed an opening into the bladder or alimentary tube beyond reach, and do not completely empty themselves through it. When such is the condition, rather than cut through solid tissue by the vagina or rectum, we must, if the condition of the patient be critical, have recourse to laparotomy. But making an outlet through the abdominal walls is of questionable benefit, unless accompanied by the more important part of the operation, viz.: the obliteration of septic pockets and establishment of perfect drainage through the vagina or rectum. But there are some abscesses producing serious symptoms before pointing below or externally, which were originally, or have become, abdominal or supra-pelvic in character. These, according to the latest experience, should unquestionably be evacuated by means of laparotomy, whenever they cannot be reached extra-peritoneally. But a large majority of them are hæmatoceles, very many of which could and should have been operated upon from below, before a threatening abdominal abscess had developed. Therefore, under proper and timely treatment from the beginning of the attack, but few cases of pelvic abscess would require laparotomy.

PROFESSOR W. H. BYFORD wished to make two or three remarks with regard to certain abscesses which had not been touched upon during the evening, viz.: such as do not break, do not discharge, but remain as chronic cavities for a number of years. He had had an opportunity of tracing three such cases for twenty-three or four years, and in each of them there had been found serous in place of pus cavities. He accounted for it in this way: the lining membrane is an ulcerated surface, and he thought that in some of these cases instead of the pus being discharged these surfaces underwent cicatrization and that there was then a cicatrized cavity in which there was an amount of serum that dissolved the pus corpuscles and thus causing their disappearance so as to leave nothing but a clear collection of serum—a serous sac. In the three cases mentioned, when the sac was opened it was found to contain serum instead of pus. He had been very much interested in them and followed them up until he had proved to his satisfaction that this change actually did take place; a cicatrization of the whole lining membrane, converting it into a non-suppurating cavity containing serum.

PROFESSOR A. REEVES JACKSON said, replying to the questions that had been asked in regard to those cases in which an opening of the abscess was already established, and when the health of the patient was not unfavorably influenced by the discharge, he would let the woman alone unless he could so enlarge the opening as to establish a thorough drainage. And in cases in which there was no opening and in which there seemed to be evidence of the existence of pus, he would endeavor to find it with the aspirator needle introduced through the vagina or abdominal wall in preference to opening through the rectum. In case drainage could be established perfectly through the vagina and abdominal wall he would rely upon that. Professor Jackson said that he had failed to make himself understood clearly in regard to the estimate he placed upon the treatment of the early stages of pelvic cellulitis. He approved of the vaginal douche, hot applications over the epigastrium, and opium, the latter being his sheet anchor, more particularly in peritonitis than in cellulitis. He thought that in many cases by lessening the pain the pulse was reduced and the temperature lowered. He simply felt that these methods of treatment do not reach the bottom of the difficulty, but in the absence of anything better he had always used them. He had never used leeches but thought they might be beneficial. The blood is not withdrawn directly from the congested vessels, but its loss may affect the part as would venesection in the arm or elsewhere. In conclusion, in a case of pelvic abscess, when a woman was dying from rapid pulse, high temperature and the constitutional disturbance that these things always bring in their train, other means having been tried unsuccessfully, he would perform laparotomy, because in many of these cases, even if the operation should fail, the patient is not made materially worse. He had come to believe that constant invalidism is not preferable to death.

III.—PROFESSOR DELASKIE MILLER reported a

*Case of Epithelioma of the Uterus; Treated by Mercuric Nitrate; Result Six Years After Treatment.*

This case derives its interest from the extent of the disease, the treatment, and the condition of the patient since treatment and now.

Mrs. R., wife of a physician, came under my care about eight years ago, affected with epithelioma of the uterus. When the case was first examined, a considerable portion of the cervix had disappeared and the disease had extended to the *os internum.* The hæmorrhage had been frequent and severe, and the ichorous discharge was constant. The general health had been greatly impaired.

*Treatment.*—Granulations were removed by the curette and nitric acid was applied. Tonics and a generous diet were added.

After a few weeks she left the city for her home, feeling better.

She again visited the city three months later. The disease had made further inroads upon the tissues of the uterus, and upper part of the vagina. Chloride of zinc was applied and reapplied. Apparent improvement followed.

The patient came to the city at intervals for about two years, and submitted to active treatment for several weeks each visit. Still the disease evidently advanced, though the symptoms were occasionally suppressed. The vaginal portion of the cervix had quite disappeared. The cavity of the uterus became involved, and the disease was extending over the upper part of the vagina, both anteriorly and posteriorly. The appetite, digestion and general strength were impaired to that degree that it was manifest the patient would succumb unless the disease could be promptly arrested.

The acid nitrate of mercury was chosen as the agent, and in the presence of Professor Jackson it was applied in the following manner: A roll of cotton two and a half inches in length, and as large as the canal would admit, was saturated with the acid nitrate of mercury and introduced, reaching into the cavity of the uterus and filling the cervical canal, and a small portion left dependent into the upper portion of the vagina. This application was left undisturbed *in situ* for seventy-two hours, and undoubtedly it is to this fact that the result is due.

After the cotton was removed an extensive slough came away. The subsequent granulations appeared healthy, and in two weeks the patient left the city, since which time I have not seen her.

This last application produced more than a local effect, viz., a more severe ptyalism than that which affected this patient it would be difficult to imagine, and this was attended by an intensely jaundiced appearance of the entire surface of the body.

The following extracts are from a letter received a few weeks ago from the husband:

"*My Dear Doctor:* I received your letter a few days ago; must acknowledge my carelessness in not writing to you; have often thought I ought to write to you in reference to my wife's condition and state of health since her last treatment. The current of time has borne us along on smooth waters, all enjoy reasonable health, peace and prosperity. Mrs. R., during the past four years, with the assistance of my daughter, has performed the duties of a housewife, with all the cares and work, except when she has been laid up for a day or two with sick headache. She has it to-day the second time in eight weeks; am giving her a new remedy which has increased the intervals between the attacks two weeks. I do not think the ulceration has ever appeared since the last treatment; have not examined her with speculum for several years. The uterus rests upon the perineum and seems heavier than it ought to be, and of late has been the cause of much disturbance of the rectum, requiring its replacement. She suffers some pain occasionally, and the menses are sometimes very profuse. There has not been any discharge of water since you applied the nitrate of mercury. It was heroic treatment, but I believe it saved her life, and I shall ever feel grateful to you and Professor Jackson for your kindness towards Mrs. R. and myself."

W. W. JAGGARD, M. D.,
2330 Indiana Ave., April 12th, 1886. *Editor.*

